# King's College Hospital

**Published:** 1903-10-17

**Authors:** 


### The Hospital.

A Journal of

The Medical Sciences and Hospital Administration.

Vol. XXXV.—No. 890. OCTOBER 17, 1903.

### King's College Hospital.

The decision arrived at by the assembled Governors of King's College Hospital on Monday, authorising the committees concerned to take the necessary steps for obtaining an Act of Parliament to sanction the removal of the hospital to some other locality, is one which unquestionably merits the support and approval of the public. It has been well known for many years, to all who are conversant with such questions, that the buildings of the hospital are exceedingly defective when estimated by the standard of modern requirements; and that, from considerations of cost and of site, as well as from the pressure of surrounding structures, they did not admit of being brought into a satisfactory condition. Those who have good memories will recall a controversy which raged several years ago with regard to the nursing arrangements of the hospital, in the course of which it was stated that there was a certain bed, close to an open window giving upon a dustheap, the occupant of which always died after an operation. The statement may possibly have been only *ben trovato*, but it served to illustrate the sanitary difficulties of the site, and its essential unfitness for the purposes to which it was applied. At that period, however, no question of the needs of the surrounding population could have been raised. The network of narrow streets in the vicinity of Drury Lane and Covent Garden was crowded with poor, consisting to a great extent of persons engaged in the more precarious forms of industry, such as street hawking and the like, of whom it has been said that three consecutive wet days bring them to the verge of starvation. The hospital, even if it were in some respects structurally defective, was at least a thousand times better than the homes from which a large proportion of the patients were derived; and there could be no doubt that it was, on the whole, a powerful influence for good in its locality. In addition to the claims upon the public thence arising, it was in successive years the scene of the teaching and of the triumphs of Liston, of Ferguson, and of Lister; of Budd, of Todd, and of Watson; and its school became a centre from which some of the most distinguished men of our time went forth to carry the latest developments of the healing art to all quarters of the globe.

Upon the conditions which formerly existed in the locality the London County Council has descended like a fairy in a pantomime, although whether entirely like a beneficent one, we will not undertake to say. However this may be, Springardenia has waved her wand, and slum dwellings are about to be superseded by palatial structures, in which the eye of the progressive councillor fondly recognises unlimited possibilities of "betterment." The surrounding poor of ten years ago are being displaced, let us hope into model dwellings to be built for them elsewhere, and will no longer have their chief centre of overcrowding within a stone's throw of the Strand. In old Rome the "proletarius" was a citizen whose condition was so mean that the only service he could render to the State was to beget children for her defence; and, in the vicinity of Drury Lane, even this service is no longer being performed as once it was. The statement issued by the Council, in advocacy of the proposed change, said that the maternity cases available for the instruction of students had fallen off in 10 years from 684 to 389. The in- and out-patients are no longer chiefly derived from the parish of St. Clement Danes, in which the hospital is situate, but almost entirely from other parts of the county of London, and chiefly from its southern districts. The immediate necessities of the locality, such as they are, can be amply served by Charing Cross Hospital; and it has long been recognised that the constant growth of London, and the regularly progressive dispersion of the labouring classes over its suburban area, call imperatively for a change in the conditions under which its hospital accommodation is provided. The document originally issued by the Council of King's College, and by the majority of the managing committee of the hospital, called forth a very feeble and ill-considered protest from the minority of the latter body, headed by Lord Dillon, the chairman; and at the meeting on Monday there was an equally feeble and ill-considered attempt to prevent or delay the inevitable change. One of the opponents suggested waiting for two years, lest it should turn out that the future inhabitants of Aldwych and Kingsway would, after all, be persons in need of hospital accommodation; and another was of opinion that the present was not a good time for incurring the inevitable expense, because "many hospitals were now begging for money." *Rusticus expectat dum defluat amnis*, and the time when other hospitals are not begging for money is hardly likely to arrive while the objectors on this score are still active in good works. The resolution in favour of removal was originally that powers should be obtained to purchase a site elsewhere; but this, on the amendment of Mr. McHardy, was altered from "purchase" to "acquire," thus wisely giving greater freedom of action to the committees concerned. It is said in a general way that the new site is to be in "South London," but no part of the extensive district thus designated has so far been mentioned in public, although it is said that more than one possible position has been under consideration. The committees estimate the cost of the new buildings at £300,000, and they propose to seek this amount from the public, and to retain the present site as a source of income for the future. We wish them every success in an undertaking eminently calculated to promote both the treatment of the sick and the progress of medicine and surgery.

